# Southern Dental Association

**Published:** 1884-07

**Authors:** 


### SOUTHERN DENTAL ASSOCIATION.

SIXTEENTH ANNUAL MEETING, HELD AT LEXINGTON, KY., MAY 6, 7, 8 AND 9, 1884.

REPORTED EXPRESSLY FOR THE INDEPENDENT PRACTITIONER.

(Continued from page 327.)

Dr. Patrick said that in the mixed European races more and greater anomalies are found in individual parts than in the diverse races of Africa or Asia. The peculiarity of the human being is that he crosses with his kind. Man is of one order, and the only species of that order. He is *genus homo,* and homogeneous as well. In the herbivorous classes of animals we find special families, for the reason that they will not cross. If they do they produce *hybrids.* So there are natural reasons for the irregularities in human teeth, because the races cross and re-cross.

The President called the attention of the convention to the Coffin method of regulating teeth, not used so much on this side the Atlantic as in Europe, where he had seen numerous models. It is simply the appliance of a split plate to the roof of the mouth, to spread it, no matter how contracted. You sometimes find an arch no wider than your finger. Put in a split plate, and it will change the whole shape of the mouth, and the shape of the face. Other appliances may be used with the split plate. Dr. Coffin is using this plate very largely. He had models where the arch had been spread over half an inch, giving an entirely different expression to the face. Nothing can be found to act with so little pain. You can put this plate into a child's mouth, when you are regulating it (and you know how hard it is to keep anything in a child's mouth), and it is never troubled. It is kept up by fitting every tooth; so the arch has nothing to interfere with it. The teeth are enclosed with a rubber plate. You can make a combination of this plate with this wire (Patrick's) in regulating teeth. Dr. Coffin has been very successful with it.

He (Dr. McKellops) tried it on his own son, going back to London to have the boy operated upon. On shipboard the lad was very sick, and would take the plate out and put it back. By the time they landed the displaced lateral incisor was in its proper place. In nine days the tooth was moved, and is there to this day. As soon as it got over the under tooth it remained there—it had to stay put.

Dr. J. Hooper, Louisville, said he has had some twenty-five cases, and has had remarkable success. In the case of one lady patient he expanded the arch nearly an inch. She is a musician in a church, and when she went to sing she took the plate out, and put it back again when she was done. Another was a case of a crossed canine tooth, which was pushed in place in seven weeks. There was no pain, except just a little soreness for the first four or five days.

The President said Dr. Coffin was of the same opinion as Dr. Patrick, that appliances can be put on at any age. He does not hesitate to put them on a person fifty years old.

Dr. Patrick described a case of irregularity by supernumerary teeth, from the model. There was a supernumerary between the left superior front incisor and the lateral. The canine had developed, and caught the gum away up above, and was crowded out and protruded over the jaw. He explained his operations, extending over a period of two years, and how he finally got the central incisor straight, but lost the canine, which he believed he could have got in place had he not been in such a hurry to extract. Some cases cannot be very well regulated, as when the irregularity arises in the form of the maxillary bones. He had models in which there are only the two front incisors; another with only the lateral incisors; others have no incisors at all, and in two of them there is hare lip. The maxillary bone may show retarded development, or it may be so developed as that the front incisors will be too short to occlude with the others. He did not see any means of regulating such cases without taking out the molars, when the bicuspids would antagonize. Arrested development may be on one side or the other, superior or inferior, on the right or left, or in the center. When the lower teeth project over the upper, if these teeth in front show a puffy border of skin, the fangs are not right, and if you attempt to move them you will move them out entirely.

Prof. R. B. Winder questioned the influence of the crossing of races as laid down by Dr. Patrick. He had studied the large collection of half-breed skulls at Washington, and in every case they are the skulls of the white man, and not of the Indian. The correct theory regarding the crossing of races is supported by skin developments. When any of the inferior animals are crossed, the progeny retains chiefly the characteristics of the male. Take for an instance that most common of hybrids, the mule. These skulls referred to, contain the teeth of the white man, and not those of the Indian.

Dr. Patrick declared that the white man who associates with the

Indian is close to the Indian (laughter). He had never been able to find marks of distinction between a white man's tooth, taking him as a white man, and that of an Indian or negro. He could take an Australian's tooth and tell it, if it were mixed in a bushel; but he couldn't take a European's tooth and tell to what race it belonged. He had tried to, but there is such a variety in teeth of Europeans that he found it impossible.

Prof. Winder remarked that he had alluded to the arches and their arrangement.

Dr. Patrick insisted that there is such a variety in the European that one cannot tell. You can hardly tell in the skulls. Samuel George Morton, of Philadelphia, had tried, but could not establish a rule. Paul Broeck, of France, could not do it. He (Dr. Patrick) had examined these collections at Washington. What astonished him was that a member of the American Dental Association had said there was not among dentists one qualified to examine crania. If he had been in the least acquainted with anthropology, he never would have said it. It is a very difficult matter to distinguish skulls of different races. Put them together, and you will find a European skull that will match any one of them. The study of one, is a case which belongs to the surrounding state of individuals when they have been isolated. But when you take two, you cannot tell. You do not know the teeth of man until you have examined the teeth of other animals. There never was any anatomy until comparative anatomy became a study, through the influence of Cuvier and in his time.

At the close of the session, Dr. A. O. Rawls was presented with a pair of beaten silver drinking-cups, made by the President, a knife and a masher. The presentation speech was neatly made by Dr. Patrick, and Dr. Rawls appropriately responded.

SECOND DAY—MORNING SESSION.

After routine business the President again called up the subject of

OPERATIVE DENTISTRY.

Prof. J. Taft, of Cincinnati, spoke with special reference to the time when interference should be made in treating irregularities.

In many instances it is made too early. Interference should be mild before the teeth have completely developed. He thought it likely that the rule given by Dr. Patrick was as reliable as anything—to wait till the indentations or cusps on the points of the incisors are worn away. There will be calcification and formation of the roots by that time. It is well not to interfere with the permanent teeth before the age of fourteen or fifteen. They may be moved after that till fifty. Differences will be found in different cases. Sometimes a tooth can be moved easily, and with very little irritation; again the operation must be conducted with very great caution, lest permanent injury be done to the parts. When a tooth is moved to one side, a filling up on the other side must take place, if you want a fixed tooth. It is a question whether this new growth in the filled place is more susceptible to disease than is the other side. He had sometimes thought a tooth, after being disturbed, more liable to disease. Where an expansion of the arch is required, a split plate is best. The pressure is against the arch, and the teeth more nearly retain their original position. The appliance used by Dr. Patrick is very good, but the Coffin plate has more sweep of application. These two seem to embrace all that is necessary. It would be well for the profession to thoroughly understand these two appliances, then there would be very little call for anything else. That it is very easy, usually, to regulate teeth, is another reason why it should not be attempted at a very early age. An intelligent parent can correct a child's tooth by very little manipulation. He had known young people to correct the position of teeth for themselves. It was better for the dentist to direct the correction, than to make a rapid change. The time ordinarily given to the correction of irregularities will yield a far larger income to the dentist in other work, and if the patients can do the work it is better that they should. A dentist will often spend five or six months regulating a tooth, and only get forty or fifty dollars. It is one of the least remunerative parts of the practice. If the cases can be managed without this waste of time it is far better they should be.

Dr. S. S. Waters, of Baltimore, produced a model in which the front teeth all projected forward and were elongated to a most unnatural degree, and asked what could be done in the case.

Prof. Taft thought it could be very much improved by pressing the teeth up a little, and throwing the roots out a little.

The President asked if the molars could not be elongated.

Prof. Taft said it might be done, but thought it as easy to do the other. He would trim the corners of the teeth.

Dr. Patrick said the alveolar process had advanced, the septums and cells all being moved forward. To be successful the operator would have to move the bicuspids back, one by one, and hold them there as they were moved. In the meantime further progress forward might be stayed, as it would increase with age. The deformity had been partly produced by a fault in the maxillary bone—the anchylosis of a suture or the non-anchylosis of another suture, as seen in the different shapes of the head. Teeth which depend on the anchylosis of bones follow the bones. The molars were not moved. Anchoring to the molars, the others could be drawn back one at a time, and when got back bound there. That would prevent them shifting forward. They would be longer. The back ones would have to be drawn up, or the others would be too long. When a tooth begins to shift, stop it, and retain it in place till a callus is formed. One might begin to move these teeth and the condition of the patient be such that no callus would form. The teeth could be brought in and fixed. If allowed to remain they would get worse; not only the incisors, but the bicuspids.

Dr. Waters, in answer to a question, stated that he had not decided what to do in the case. He merely had it under consideration. The guardian of the patient had almost decided what should be done.

The President and others favored pulling all the front teeth out.

(TO BE CONTINUED.)

